# Clinical Outcomes of Surgery Versus Radiotherapy in Bilsky Grade 3 Metastatic Epidural Spinal Cord Compression

**DOI:** 10.3390/jcm15010216

**Published:** 2025-12-27

**Authors:** Kihyun Kwon, Sehan Park, Myeong Geun Song, Wan Soo Park, Chang Ju Hwang, Dong-Ho Lee, Jae Hwan Cho

**Affiliations:** Department of Orthopedic Surgery, Asan Medical Center, University of Ulsan College of Medicine, Seoul 05505, Republic of Korea; 899835@naver.com (K.K.);

**Keywords:** Bilsky grade 3, metastatic epidural spinal cord compression, radiation therapy, surgery, spinal metastases

## Abstract

**Background/Objectives**: Surgery is generally recommended for higher Bilsky grade metastatic epidural spinal cord compression (MESCC); however, Bilsky grades 2–3 are often grouped together, leaving limited evidence for managing patients with Bilsky grade 3 MESCC who have not developed neurological deficits. This study aimed to evaluate whether, and when, surgery should be performed in Bilsky grade 3 MESCC. **Methods**: This retrospective cohort study included patients diagnosed with Bilsky grade 3 MESCC from January 2021 to January 2025. A total of 138 patients were assigned to a radiotherapy (RT) group (*n* = 54) or a surgery group (*n* = 65) based on initial treatment. Demographics, clinical data, treatment outcomes, and treatment modalities were analyzed. Logistic regression identified risk factors for local progression, motor recovery, and ambulatory outcomes. **Results**: Ninety-five patients (70.3%) initially presented with weakness. Among 30 patients diagnosed before neurological deficits, interval from diagnosis to onset was 17.2 ± 14 days. Local progression and survival rates did not significantly differ between the groups. Surgery was associated with a higher likelihood of motor recovery (odds ratio [OR] = 10.05, *p* < 0.001) and better ambulatory function (OR = 0.433, *p* = 0.003). Higher initial motor grade and lower Eastern Cooperative Oncology Group Performance Status scores were also linked to favorable ambulatory outcomes. **Conclusions**: In Bilsky grade 3 MESCC, the mean interval from diagnosis to weakness onset was 17.2 days. Local progression and survival did not differ between RT and surgery; however, surgery provided superior motor recovery and ambulatory outcomes. Early surgery may offer improved functional outcomes in Bilsky grade 3 MESCC.

## 1. Introduction

Metastatic epidural spinal cord compression (MESCC) imposes a substantial clinical burden on patients and often results in severe pain, neurological deficits, and multiple comorbidities [1]. Advances in radiotherapy (RT) have broadened treatment options for MESCC [2,3]. Lower Bilsky grade MESCC is typically managed effectively with RT alone [4], whereas surgery is generally recommended for higher-grade disease because of neurological risks [5].

Current guidelines, including the Neurologic, Oncologic, Mechanical, and Systemic (NOMS) framework and the Neurology-Stability-Epidural compression (NSE) assessment, do not differentiate between Bilsky grades 2 and 3 [6,7]. A recent study by Park et al. reported that RT yielded favorable local control in Bilsky grade 2 MESCC [8], prompting questions regarding appropriate management of Bilsky grade 3 MESCC. However, few studies have focused specifically on patients with Bilsky grade 3 MESCC. Delayed surgery has been associated with poorer recovery of ambulatory function in patients with neurological deficits [9], and prompt treatment can help minimize the duration of neurological compromise [10]. However, for patients without neurological deficits, evidence on whether and when surgery should be performed remains limited.

Therefore, we conducted a retrospective cohort study of patients with Bilsky grade 3 MESCC to identify the therapeutic window for patients presenting without neurological symptoms and to evaluate treatment approaches for local control and ambulatory outcomes.

## 2. Materials and Methods

### 2.1. Study Design and Patient Inclusion

After institutional review board approval (IRB: 2025-0812), we conducted a retrospective cohort study of patients diagnosed with Bilsky grade 3 MESCC between January 2021 and January 2025. We searched our institutional data warehouse for magnetic resonance imaging interpretations containing “Grade 3,” “ESCC 3,” “MESCC 3,” or “Bilsky 3.” All interpretations were made by board-certified radiologists specializing in musculoskeletal imaging. An experienced board-certified orthopedic surgeon subsequently reviewed all images and excluded patients with inconsistent interpretations. Additional exclusion criteria were (1) MESCC below the conus medullaris, (2) a follow-up period <3 months, and (3) insufficient clinical data. Patients who died before 3 months were included.

### 2.2. Data Collection and Outcome Evaluation

Demographic variables included age, sex, comorbidities such as diabetes mellitus (DM) and hypertension (HTN), and smoking status. We also collected data on primary tumor pathology, Eastern Cooperative Oncology Group Performance Status (ECOG), and ambulatory ability. Baseline ECOG was defined as the patient’s usual functional activity prior to the onset of neurological deterioration, in order to reflect pre-morbid systemic condition rather than disability related to acute cord compression or pain. ECOG at presentation and after treatment was also recorded but was not used as the primary variable for analysis. ECOG assessed after neurological deterioration was not used for baseline comparison, as it was frequently influenced by acute motor weakness. For further analysis, primary tumor pathologies were categorized according to radiosensitivity into three groups: radiosensitive, radioresistant, and intermediate. This classification was based on prior literature [11]. Tumors known to be radiosensitive or radioresistant were assigned accordingly, and those not clearly belonging to either category were allocated to the intermediate group. Imaging data included tumor level, compression direction, presence of pathologic fracture, and Spine Instability Neoplastic Score (SINS). Treatment decisions after diagnosis of Bilsky grade 3 MESCC were reviewed, and patients initially treated with RT were classified into an RT group, whereas patients treated surgically were assigned to a surgery group. Decisions regarding initial treatment modality were reached through multidisciplinary consensus among oncologists, spine surgeons, and radiation oncologists. Treatment allocation incorporated several clinical factors, including baseline ECOG performance status, SINS, comorbidity burden, estimated life expectancy, prior radiotherapy to the involved level, anatomical feasibility of decompression and stabilization, and patient preference. Patients who were initially evaluated as potential surgical candidates but ultimately did not undergo surgery, due to systemic deterioration, comorbidities, or preference, were analyzed in the radiotherapy group. Patients who did not receive any active treatment or received only chemotherapy were included in the dataset for descriptive purposes but were excluded from comparative analyses. Treatment modality information, including surgical approach and technique, RT modality, external beam radiation therapy (EBRT) or stereotactic body radiation therapy(SBRT), RT dosage per fraction, and total dosage, was collected.

Outcome measures included local progression, motor recovery, and ambulatory function. Local progression was defined as tumor progression occurring after treatment that required further intervention at the same anatomical level. This determination was made based on a combination of clinical symptoms and follow-up MRI findings. Motor strength was evaluated using the modified Medical Research Council (mMRC) scale; the lowest recorded strength was used for analysis. Improvement in the same myotome was considered recovery; unchanged strength was considered maintained, and decreased strength was considered worsened. Logistic regression analyses excluded patients who had maintained grade 5 motor strength throughout. Motor recovery was categorized as favorable, whereas maintained or worsened strength was classified as an adverse outcome. Ambulatory function was assessed by the ability to walk; preserved or restored walking ability was considered success, whereas loss of ambulation was considered failure. Post-treatment imaging was not performed at fixed intervals. Follow-up MRI was obtained when patients developed new or worsening symptoms, or when clinical reassessment raised concern for progression. This symptom-driven imaging approach was applied similarly in both the surgery and radiotherapy groups.

### 2.3. Statistical Analyses

Statistical analyses were performed using SPSS version 24 (IBM Corp., Chicago, IL, USA) and Python version 3.11.8 (statsmodels 0.13.5, scikit-learn 1.1.3). A two-sided *p*-value < 0.05 was considered statistically significant. Student’s *t* test or Mann–Whitney U test was used for continuous variables, and the chi-square test was used for categorical variables. Logistic regression was used to identify risk factors for the outcomes. Kaplan–Meier survival analysis was performed to compare survival between the two groups.

## 3. Results

**Figure 1:**
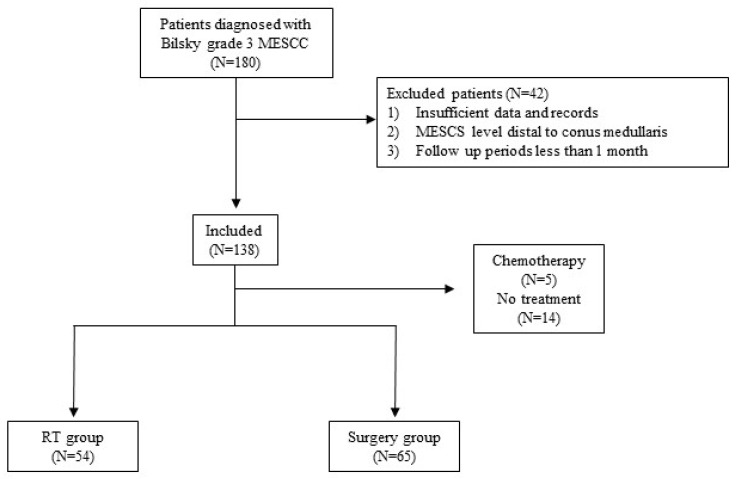
Flow chart of patient selection process.

**Figure 2:**
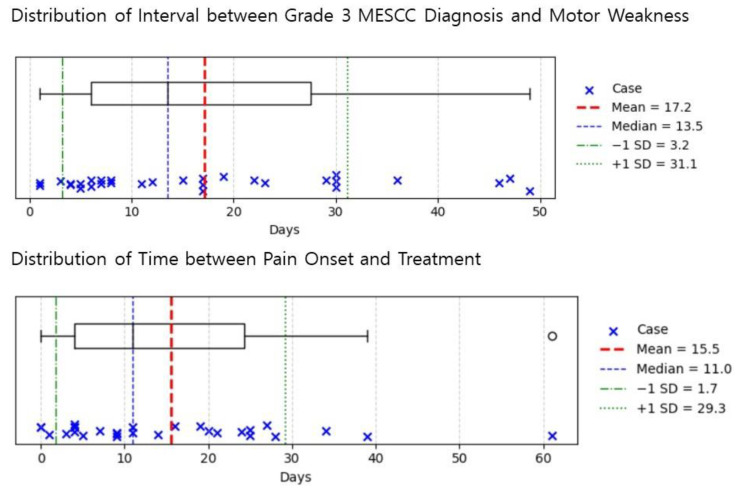
Distribution of the interval from diagnosis to onset of motor weakness (**top**) and the interval from pain onset to treatment initiation (**bottom**).

After applying the inclusion and exclusion criteria, 138 patients were included (Figure 1). Fifty-four patients (39.1%) received initial RT, and 65 (47.1%) underwent surgery. Five patients (3.6%) received chemotherapy, and 14 (10.1%) chose conservative management. Among the 95 patients (70.3%) who presented with weakness, 30 (31.6%) were diagnosed before weakness developed. The mean interval from diagnosis to onset of weakness was 17.2 ± 14 days (Figure 2 top). Although a few patients developed weakness after more than 40 days, most had an intervention window of less than 30 days once diagnosed with Bilsky grade 3 MESCC. Patients who initially presented with pain were treated within 15.5 ± 13.8 days (Figure 2 bottom). Two patients presented with pain alone and began RT 29 and 30 days after diagnosis, but later developed weakness.

### 3.1. Comparison Between the RT and Surgery Groups

**Figure 3:**
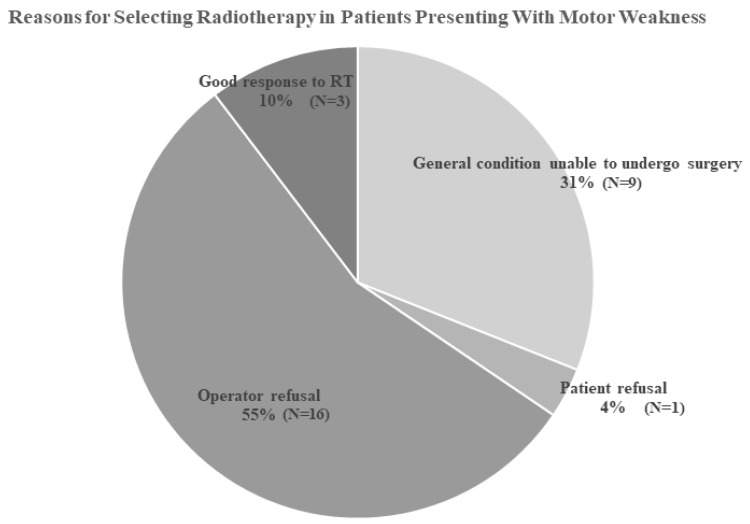
Pie chart illustrating the reasons for selecting radiotherapy over surgery in patients presenting with motor weakness.

We compared patients treated with RT with those treated surgically (Table 1). Demographic characteristics were not significantly different between the groups. Initial symptoms varied, and weakness was more common as a presenting symptom in the surgery group. The average motor grade was also more severe in the surgery group than in the RT group. Other parameters, including the ECOG status, involved location, presence of pathologic fracture, direction of compression, and SINS, did not differ significantly. We examined reasons for selecting RT instead of surgery for patients presenting with weakness (Figure 3). In 36% of patients, surgical procedures and general anesthesia were not feasible. In 55% of patients, the surgical department elected not to perform surgery despite referral. Ten percent of patients had a favorable response to RT, and 4% declined surgery.

**Figure 4:**
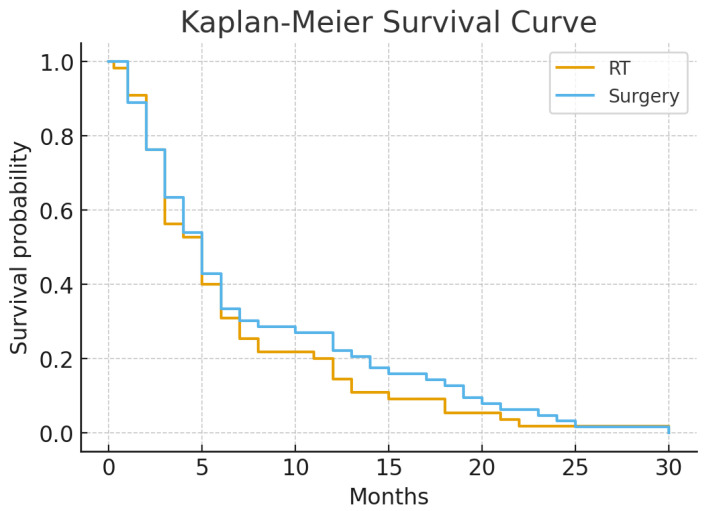
Kaplan–Meier survival curves comparing overall survival between the radiotherapy and surgery groups.

Table 2 presents the comparison of clinical outcomes between the groups. Motor recovery and ambulation recovery rates were significantly higher in the surgery group. More patients in this group demonstrated improved or stable motor strength, whereas a higher proportion of patients in the RT group experienced worsened motor strength after treatment. Local progression rate, survival duration, and complication rate did not differ significantly. Kaplan–Meier survival analysis showed no difference between the groups (Figure 4).

**Figure 5:**
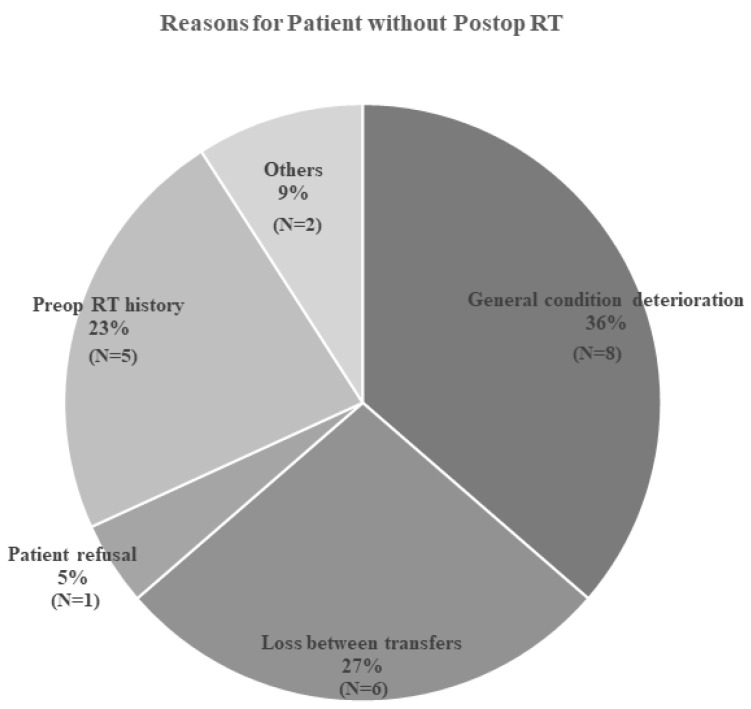
Pie chart illustrating the reasons why some patients did not receive postoperative radiotherapy after surgery.

To identify risk factors for local progression, we further analyzed patients according to specific approaches and RT modalities. Most patients in the surgery group underwent a posterior approach with laminectomy and fusion (Table 3). Among patients who received RT, 41.9% underwent SBRT, and 44.4% of patients initially treated with RT received SBRT. A relatively large proportion of patients did not undergo postoperative RT (n = 22, 33.8%). Most of these patients (n = 8, 36%) were unable to receive postoperative RT because their general condition deteriorated after surgery (Figure 5). A substantial number (n = 6, 27%) were lost to follow-up during transfers between departments or hospitals. Additionally, 23% of patients (n = 5) could not receive postoperative RT due to preoperative RT history.

When stratified by tumor radiosensitivity, local progression, ambulatory recovery, and motor improvement were comparable among the sensitive, intermediate, and resistant groups. In RT-treated patients, outcomes were likewise similar across radiosensitivity categories, none reaching statistical significance (Supplementary Tables S1 and S2).

### 3.2. Risk Factor Analysis for Local Progression, Motor Recovery, and Ambulatory Outcomes

After subcategorization, we evaluated risk factors for local progression (Table 4). DM was a significant risk factor, with an odds ratio (OR) of 3.00 (95% confidence interval [CI] 1.07–8.40; *p* = 0.04). In the surgery group, postoperative RT showed a marginal protective effect (OR 0.32; *p* = 0.06). DM was also a risk factor in the RT group. The initial treatment modality (surgery vs. RT) did not significantly influence the risk of local progression. Multivariate analysis confirmed these findings, with postoperative RT maintaining marginal significance.

Regression analysis for motor recovery was conducted (Table 5). Surgery as the initial treatment demonstrated a strong favorable association with recovery (OR = 9.82, 95% CI 3.49–27.67, *p* < 0.001). Higher SINS were linked to a greater likelihood of recovery (OR = 1.20, 95% CI 1.03–1.40, *p* = 0.02). However, in the multivariate analysis, only treatment modality remained significant (OR = 10.05, 95% CI 3.36–30.10, *p* < 0.001). Ambulatory outcomes were also analyzed (Table 6). Surgery as the initial treatment (OR = 4.33, 95% CI 1.66–11.29, *p* = 0.003) and higher initial motor power (OR = 1.57, 95% CI 1.12–2.19, *p* = 0.01) were significantly associated with better ambulation. Higher ECOG grades were associated with poorer outcomes (OR = 0.60, 95% CI 0.40–0.92, *p* = 0.02). Cervical metastasis showed a trend toward better outcomes than thoracic or multilevel metastases (OR = 0.26, 95% CI 0.07–1.07, *p* = 0.05), although this was not significant in the multivariate analysis.

## 4. Discussion

In our study, we analyzed patients with Bilsky grade 3 MESCC. The average interval between diagnosis of Bilsky grade 3 MESCC and onset of weakness was 17.2 ± 14 days. Patients presenting with neurologic deficits were more often treated surgically. Local progression and survival rates did not differ between the groups; however, motor strength recovery and ambulatory function were superior in the surgery group. Treatment modality was not a risk factor for local progression, but it had a significant effect on motor strength recovery and ambulatory outcomes.

In our study, patients with Bilsky grade 3 appeared more vulnerable to neurological deficits. Among 138 included patients, 90 had motor weakness, and 60 of them showed weakness prior to diagnosis. Even in patients without clinical symptoms of neurological deficit, the safety margin was very short, averaging 17.2 days. Patients who presented with pain only were also treated within 30 days to remain free of neurological deterioration. These results differ from the finding that 67% of Bilsky grade 2 patients initially presented with pain [7]. However, prior work reported that paralysis severity was not associated with the degree of cord compression [12], our findings suggest that higher compression grades may carry a greater risk of paralysis. Therefore, we propose that patients diagnosed with Bilsky grade 3 MESCC should be considered for early surgical intervention.

Secondly, in our cohort, a substantial proportion of patients (25 of 29, 86.2%) were treated with RT rather than surgery because of comorbidities and the risk of deterioration in overall condition. Given that patients with higher Bilsky grades have poorer survival [11], previous studies have emphasized careful patient selection to limit surgical stress in frail individuals [13]. In our cohort, survival and complication rates were not significantly different between the two groups. Even among patients with limited life expectancy, quality of life should remain a key factor when considering a surgical intervention [14]. Therefore, if survival outcomes are comparable between surgery and RT, surgical treatment may be recommended more strongly. However, as this was a retrospective cohort study, patients in better general condition may have been more likely to receive surgery, whereas those in poorer condition may have been directed toward RT. A small number of patients who were initially considered for surgery but ultimately treated with radiotherapy due to clinical deterioration were included in the radiotherapy group (n = 3). Although this subgroup was very small relative to the overall cohort, its inclusion may still contribute to residual confounding, and this should be considered when interpreting the comparative results. Baseline ECOG performance status was comparable between the two groups. Nevertheless, ECOG alone may not fully capture differences in systemic fitness, and residual selection bias is likely to persist. Additional studies are needed to further clarify these observations.

The local progression rate did not differ between the groups, and neither surgery nor RT was a risk factor for local progression. In our study, only DM was associated with local progression. Previous reports have shown that DM contributes to immune dysfunction, which may worsen outcomes in metastatic cancer, aligning with our findings [15,16,17]. However, the small sample size introduces the possibility of confounding factors that require further evaluation. Although postoperative RT demonstrated a marginally significant protective effect, this result is consistent with the findings of Hu et al. [2]. Despite the established importance of postoperative RT for improved outcomes, many patients were lost during intra- or inter-hospital transfers, preventing them from receiving postoperative RT. To reduce the risk of recurrence, enhanced communication across transfer settings is essential.

The SINS is recognized as an important determinant when selecting surgery or RT [18,19]. However, most patients with Bilsky grade 3 MESCC in our cohort had a high SINS, which limited its significance in the analysis. The radiation modality also did not significantly influence progression. Although SBRT showed a numerically lower rate of local progression compared with EBRT, this difference did not reach statistical significance. Wong et al. similarly reported no difference in local progression between EBRT and SBRT [20,21], which is consistent with our findings. Radiation dose per fraction was also not associated with local progression in our cohort, despite prior reports suggesting its importance [22]. This may reflect the small sample size of the SBRT subgroup and the consequent limited ability to adjust for confounders such as radiosensitivity, tumor biology, and prior RT exposure. Therefore, the absence of a statistically significant difference between SBRT and EBRT should be interpreted with caution. Accordingly, no definitive conclusions regarding the comparative efficacy of EBRT versus SBRT can be drawn from this dataset.

Motor strength and ambulatory recovery were superior in the surgery group. Although higher SINS values were associated with improved recovery in the univariate analysis, this association was not significant in the multivariate analysis. This finding likely reflects that patients with higher SINS values were more likely to undergo surgery, suggesting confounding between SINS values and treatment modality. When categorized by ambulatory function, ECOG score and initial motor power were significant predictors of recovery. Because ECOG performance status reflects ambulatory capacity, and motor grade is essential for ambulation, these results align with our expectations. Although surgery intuitively offers greater potential for motor and ambulatory improvement because direct spinal cord decompression is more effective than RT [23,24], few studies have evaluated these outcomes specifically in patients with Bilsky grade 3 MESCC.

Because patients with Bilsky grade 3 MESCC face a high risk of rapid neurologic deterioration, and given the superior functional outcomes observed with surgery along with comparable comorbidity profiles, survival rates, and local progression rates, we propose surgery as the preferred treatment for patients with or without weakness.

The main limitation of this study is its retrospective design, which prevents complete exclusion of selection bias in the treatment choice. In addition, the number of patients was too small for meaningful subgroup analyses or identification of additional risk factors. For instance, we could not differentiate tumor pathologies, although distinct pathologies may yield different outcomes. Variations in radiosensitivity based on primary pathology may substantially influence treatment results [25]. Hepatocellular carcinoma showed a higher local progression rate than other pathologies in our cohort, but the sample size (22 cases) was insufficient for statistical significance. Large, multi-center studies are necessary to obtain more comprehensive data on the relatively uncommon Bilsky grade 3 MESCC and to validate the findings of this study.

## 5. Conclusions

In patients diagnosed with Bilsky grade 3 MESCC, the mean interval to weakness onset was 17.2 days. Local progression and survival did not differ between RT and surgery. Surgical treatment was associated with better motor recovery and ambulatory outcomes. Timely surgical intervention in Bilsky grade 3 MESCC may offer better functional results in appropriately selected patients. Further prospective studies are needed to validate these findings. 

## Figures and Tables

**Table 1:** Comparison of demographics between RT and Surgery group.

	RT Group (N = 54)	Surgery Group (N = 65)	*p*-Value
**Patient demographics**			
Age	61.62 (±11.86)	58.12 (±12.41)	0.124
Sex (M:F)	34:20	48:17	0.235
DM	8	12	0.615
HTN	20	13	0.208
Smoking	22	27	1.000
**Pathology**			0.301
Lung	17	16	
HCC	10	12	
RCC	2	5	
Breast cancer	2	3	
GI	6	10	
Hematologic	4	4	
GU	9	3	
OBGY	2	4	
Sarcoma	2	3	
Others	0	5	
**Clinical symptoms**			
ECOG	2.10 ± 1.22	2.12 ± 1.09	0.895
Initial symptoms			0.003 *
No symptom	2	0	
Pain	23	14	
Weakness	29	51	
Motor grade for patients with weakness	3.54 ± 1.72	2.90 ± 1.54	0.042 *
**Image findings**			
Involved location (Cervical:Thoracic:Multiple)	6:45:3	8:56:1	0.499
Pathologic fracture	24	42	0.063
Compression direction (Anterior:Posterior:Circumferential)	5:6:43	10:7:48	0.673
SINS	10.27 ± 3.23	11.08 ± 2.84	0.16

RT: Radiotherapy; M: Male; F: Female; DM: Diabetes Mellitus; HTN: Hypertension; HCC: Hepatocellular carcinoma; RCC: Renal cell carcinoma; GI: Gastrointestinal; GU: Genitourinary; OBGY: Obstetrics and gynecology; ECOG: Eastern Cooperative Oncology Group Performance Status; SINS: Spine Instability Neoplastic Score. * *p*-value < 0.05.

**Table 2:** Outcome comparison between RT and Surgery group.

	RT Group (N = 54)	Surgery Group (N = 65)	*p*-Value
**Motor recovery**			<0.001 *
Improved	6	32	
Maintained	24	27	
Worsened	24	6	
**Ambulation recovery**			0.017 *
Success	21	40	
Failure	33	25	
**Local progression**	11(20.3%)	15 (23.1%)	0.825
**Survival period**	6.31 ± 6.01	7.55 ± 7.17	0.600
**Complications**	2	5	0.454

RT: Radiotherapy. * *p*-value < 0.05.

**Table 3:** Treatment approaches and modes for both Surgery and RT patients.

	**Patient Treated with Operation**
Operation Approach	
Anterior	5 (7.7%)
Posterior	57 (88.6%)
Combined	3 (4.6%)
Operation type	
Laminectomy	2 (3%)
Laminectomy and fusion	59 (90.8%)
Corpectomy	4 (6.2%)
Postop RT	
Yes	43 (66.2%)
No	22 (33.8%)
RT type	
EBRT	25 (58.1%)
SBRT	18 (41.9%)
	**Patient treated with Radiotherapy**
RT type	
EBRT	30 (55.6%)
SBRT	24 (44.4%)

RT: Radiotherapy; EBRT: external beam radiation therapy; SBRT: stereotactic body radiation therapy.

**Table 4:** Regression analysis of risk factors for local progression.

All Patients	Univariate OR (95% CI)	*p*-Value	Multivariate OR (95% CI)	*p*-Value
Age	0.99 (0.96–1.03)	0.73		
Sex (F)	0.60 (0.22–1.65)	0.32	—	—
DM	3.00 (1.07–8.40)	0.037 *	2.89	0.050 *
HTN	0.55 (0.19–1.61)	0.28	—	—
Smoking	1.06 (0.44–2.56)	0.89	—	—
Pathology	0.82 (0.39–1.73)	0.36	—	—
ECOG	0.99 (0.68–1.45)	0.96	—	—
Initial motor power	0.90 (0.69–1.17)	0.44	—	—
Tumor Location (Cervical, Thoracic, Multiple)	1.12 (0.79–1.59)	0.52	—	—
Pathologic Fx	1.00	0.09	—	—
Compression direction (Ant, post, circumferential)	1.00	0.33	—	—
SINS total score	0.98 (0.85–1.13)	0.81	—	—
Initial Tx (Surgery vs. RT)	1.17 (0.49–2.82)	0.72	—	—
RT modality (SBRT)	0.89 (0.35–2.23)	0.80	—	—
**Surgery Group**				
Age	0.99 (0.94–1.04)	0.64	—	—
Sex (F)	0.64 (0.16–2.63)	0.54	—	—
DM	1.91 (0.48–7.52)	0.36	—	—
HTN	0.49 (0.10–2.47)	0.39	—	—
Smoking	1.86 (CI 0.58–5.97)	0.29	—	—
ECOG	1.03 (0.60–1.75)	0.92	—	—
Initial motor grade	0.82 (0.56–1.20)	0.31	—	—
Tumor Location (Cervical, Thoracic, Multiple)	—	0.49	—	—
Pathologic Fx	1.12 (0.33–3.81)	0.85	—	—
Compression direction (Ant, post, circumferential)	—	0.13	—	—
SINS total	0.99 (0.81–1.21)	0.90	—	—
Postoperative RT	0.32 (0.10–1.06)	0.061 *	0.29 (0.08–1.03)	0.055 *
Surgery type	—	0.73	—	—
**RT Group**				
Age	1.003 (0.95–1.06)	0.92	—	—
Sex (F)	0.57 (0.13–2.47)	0.46	—	—
DM	5.57 (1.12–27.66)	0.036 **	5.44 (1.02–29.00)	0.047 **
HTN	0.63 (0.15–2.74)	0.54	—	—
Smoking	0.47 (0.11–2.03)	0.31	—	—
Pathology	—	0.21	—	—
ECOG	0.95 (0.55–1.64)	0.86	—	—
Tumor Location (Cervical, Thoracic, Multiple)	—	0.47	—	—
Pathologic Fx	0.76 (0.20–2.87)	0.68	—	—
Compression direction (Ant, post, circumferential)	—	0.67	—	—
SINS total	0.97 (0.79–1.19)	0.78	—	—
RT modality (SBRT)	0.66 (0.17–2.58)	0.55	—	—
Dose per Fraction	1.00 (0.994–1.006)	0.55	—	—
Total Radiation Dose	1.00 (0.997–1.014)	0.17	—	—

RT: Radiotherapy; F: Female; DM: Diabetes Mellitus; HTN: Hypertension; ECOG: Eastern Cooperative Oncology Group Performance Status; Fx: Fracture; SINS: Spine Instability Neoplastic Score; Tx: Treatment; SBRT: stereotactic body radiation therapy. * *p*-value near 0.05, ** *p*-value < 0.05.

**Table 5:** Regression analysis of risk factors for Motor recovery.

All Patients (N = 80)	Univariate OR (95% CI)	*p*-Value	Multivariate OR (95% CI)	*p*-Value
Age	1.11 (0.97–1.04)	0.812		
Sex (F)	1.00 (0.46–2.72)	0.950	—	—
DM	0.96 (0.32–2.85)	0.942		
HTN	0.56 (0.22–1.44)	0.23	—	—
Smoking	0.78 (0.34–1.80)	0.56	—	—
Pathology	—	0.71	—	—
ECOG	1.24 (0.86–1.80)	0.25	—	—
Initial motor power	1.12 (0.87–1.44)	0.39	—	—
Location (C/T/M)	0.38	0.14	—	—
Pathologic Fx	1.01 (0.43–2.36)	0.99	—	—
Compression direction (Ant, post, circumferential)	—	0.23	—	—
SINS total score	1.20 (1.03–1.40)	0.02 *	1.18 (0.99–1.40)	0.07
Initial Tx (Surgery)	9.82 (3.49–27.67)	<0.001 **	10.05 (3.36–30.10)	<0.001 **
RT modality (SBRT)	0.76 (0.31–1.86)	0.55	—	—

RT: Radiotherapy; F: Female; DM: Diabetes Mellitus; HTN: Hypertension; ECOG: Eastern Cooperative Oncology Group Performance Status; C; Cervical; T: Thoracic; M: Multiple; Fx: Fracture; SINS: Spine Instability Neoplastic Score; Tx: Treatment; SBRT: stereotactic body radiation therapy. * *p*-value < 0.05, ** *p*-value < 0.005.

**Table 6:** Regression analysis of risk factors for ambulation recovery.

All Patients	Univariate OR (95% CI)	*p*-Value	Multivariate OR (95% CI)	*p*-Value
Initial treatment Surgery vs. RT	2.33 (1.10–4.90)	0.03 *	4.33 (1.66–11.29)	0.003 **
ECOG	0.47 (0.33–0.68)	<0.001 **	0.60 (0.40–0.92)	0.02 *
Initial motor power	1.57 (1.21–1.99)	<0.001 **	1.57 (1.12–2.19)	0.01 **
Metastasis level Thoracic vs. Cervical	0.26 (0.07–1.07)	0.05 *	0.28 (0.07–1.16)	0.08
Metastasis level Multiple vs. Cervical	0.09 (0.01–1.60)	0.07	0.09 (0.01–1.56)	0.09

OR: Odds ratio; CI: Credible interval; RT: Radiotherapy; ECOG: Eastern Cooperative Oncology Group Performance Status. * *p*-value < 0.05, ** *p*-value < 0.005.

## Data Availability

The data presented in this study are available on request from the corresponding author due to ethical and institutional regulations, the data are not publicly available.

## Supplementary Materials

The following supporting information can be downloaded at: https://www.mdpi.com/article/doi/s1/.

## Abbreviations

The following abbreviations are used in this manuscript:

MESCC	Metastatic epidural spinal cord compression
RT	Radiotherapy
SBRT	Stereotactic body radiation therapy
EBRT	External beam radiation therapy
DM	Diabetes mellitus
HTN	Hypertension
ECOG	Eastern Cooperative Oncology Group performance status
SINS	Spine Instability Neoplastic Score
mMRC	Modified Medical Research Council
OR	Odds ratio
CI	Confidence interval
IRB	Institutional review board
