# Supplementary material for: Clinical Outcomes of Surgery Versus Radiotherapy in Bilsky Grade 3 Metastatic Epidural Spinal Cord Compression
Source: J Clin Med. 2025 Dec 27;15(1):216. doi: 10.3390/jcm15010216 (PMC12786538; doi:10.3390/jcm15010216)
Supplement: Supplementary file 1 [file jcm-15-00216-s001.zip › jcm-4007859-supplementary.pdf]

Supplementary Table S1. Treatment outcomes by tumor radiosensitivity (Radiotherapy + surgery)

Radiosensitivity	Sensitive (N=24)	Intermediate (N=64)	Resistant (N=31)	p-value
Local progression	5 (20.8%)	14 (21.9%)	7 (22.6%)	0.99
Ambulation success	13 (54.2%)	31 (48.4%)	17 (54.8%)	0.80
Motor improved	15 (62.5%)	33 (51.6%)	19 (61.3%)	0.53

Supplementary Table S2. . Treatment outcomes by tumor radiosensitivity (Radiotherapy)

Radiosensitivity	Sensitive (N=10)	Intermediate (N=30)	Resistant (N=14)	p-value
Local progression	2 (20.0%)	6 (20.0%)	3 (21.4%)	0.93
Ambulation success	4 (40.0%)	11 (36.7%)	6 (42.9%)	0.75
Motor improved	4 (40.0%)	10 (33.3%)	8 (57.1%)	0.47
